# Phylogenetic Diversity of *Peltigera* Cyanolichens and Their Photobionts in Southern Chile and Antarctica

**DOI:** 10.1264/jsme2.ME14156

**Published:** 2015-04-28

**Authors:** Catalina Zúñiga, Diego Leiva, Lía Ramírez-Fernández, Margarita Carú, Rebecca Yahr, Julieta Orlando

**Affiliations:** 1 Department of Ecological Sciences, Faculty of Sciences, Universidad de ChileCasilla 653. SantiagoChile; 2 Royal Botanic Garden Edinburgh20A Inverleith Row, EdinburghUK EH3 5LR

**Keywords:** *Peltigera*, *Nostoc*, phylogenetic diversity, Chile and Antarctica, *Nothofagus* forests

## Abstract

The lichen genus *Peltigera* has been mainly revised in the Northern Hemisphere, with most species being recorded in Europe and North America. This study assessed the phylogenetic diversity of the mycobionts and cyanobionts of *Peltigera* cyanolichens collected in Southern Chile and Antarctica, areas in which lichens are extremely diverse but poorly studied. The operational taxonomic units (OTUs) of each symbiont were defined by analyzing the genetic diversity of the LSU and SSU rDNA of the mycobionts and cyanobionts, respectively, and a phylogenetic approach was used to relate these OTUs with sequences previously reported for *Peltigera* and *Nostoc*. Among the 186 samples collected, 8 *Peltigera* and 15 *Nostoc* OTUs were recognized, corresponding to sections *Peltigera*, *Horizontales*, and *Polydactylon*, in the case of the mycobionts, and to the *Nostoc* clade II, in the case of the cyanobionts. Since some of the OTUs recognized in this study had not previously been described in these areas, our results suggest that the diversity of *Peltigera* reported to date in the regions studied using traditional morphological surveys has underestimated the true diversity present; therefore, further explorations of these areas are recommended.

Lichens are stable symbiotic associations between a fungus, or mycobiont, and at least one photo-autotrophic component, or photobiont, consisting of a micro-alga and/or cyanobacterium. In the latter case, they are called cyanolichens and comprise approximately 10% of the currently known lichen symbioses (10).

Approximately one fifth of all known fungi have been described as obligately lichen-forming species (19), reflecting the evolutionary success of this symbiotic association. Moreover, since the diversity of lichens has mainly been determined based on morphological characteristics, recent studies clearly demonstrated that the total number of lichen-forming fungi was underestimated due to the existence of cryptic species, which are either not evident macroscopically or have been generally overlooked and only distinguished by molecular analyses (*e.g.* 7, 23, 24). The diversity of lichen-associated photobionts has also been underestimated, with examples of cryptic and poorly-known species (20, 44, 45, 48, 53) and even deeper previously unrecognized divergences (22, 31, 35) being reported.

*Peltigera* lichens commonly occur in humid, mainly shaded habitats, on the forest floor, or along roadsides or other disturbed environments. They are predominantly terricolous or muscicolous (*i.e.*, living on soil or mosses, respectively) and are rarely saxicolous or corticolous (*i.e.*, growing or living on rocks or tree bark, respectively).

Symbiotic entities within *Peltigera* are represented by two different types of associations: (i) bipartite symbioses involving the fungus and a cyanobacterium (*Nostoc*) and (ii) tripartite symbioses involving the fungus, a green alga (*Coccomyxa*) as the main photobiont, and a cyanobacterium (*Nostoc*) located in external cephalodia on the upper or lower surface of the thallus (27). Although the genus is readily recognized in the field, it is a taxonomically complex group, and many challenges remain at the species level (13, 15, 27, 28).

This cosmopolitan genus, with an estimated number of 60–75 taxa (13, 19, 55), has been mainly revised in the Northern Hemisphere (*e.g.* 15, 29), with most species recorded from Europe and North America, but also in some parts of the Southern Hemisphere such as New Zealand (11, 12), Australia (21), Papua New Guinea (47) and, recently, Southern Chile (25, 40, 41). However, these last studies, except for that by Ramírez-Fernández *et al.* (41), were only based on a morphological classification and, therefore, the diversity of *Peltigera* in Chile may be underestimated.

Our main objective was to determine the diversity of *Peltigera* symbionts from localities of Southern Chile and Antarctica, which are very diverse regions that remain poorly-studied in lichenological terms.

## Materials and Methods

### Study sites and lichen samples

**Fig. 1:**
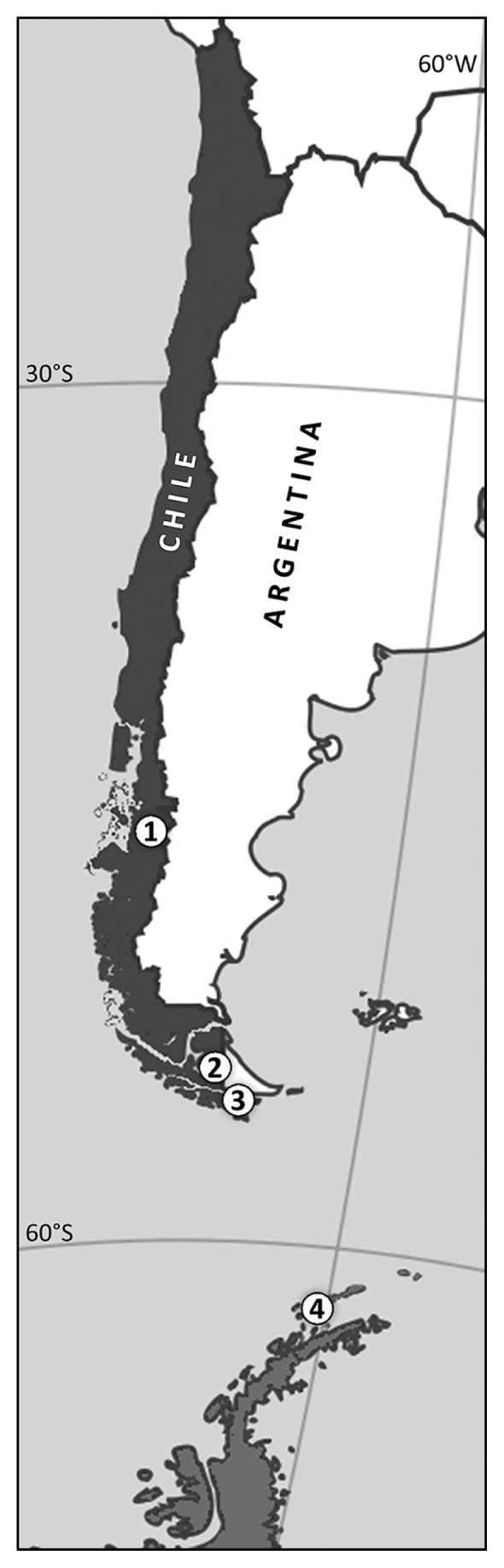
Sampling sites. The locations of the four sampling sites are shown in Arabic numbers. 1: Coyhaique, 2: Karukinka, 3: Navarino, 4: Deception.

A total of 186 *Peltigera* thalli-fragments were collected, 51 of which were from the Coyhaique National Reserve (Aysén Region, Chile; 45°31′42.96″S, 72°1′51.95″W; hereafter referred to as Coyhaique); 60 from Karukinka Natural Park (Tierra del Fuego Island, Chile; 54°07′51.67″S, 68°42′33.96″W; hereafter referred to as Karukinka); 60 from Puerto Williams, (Navarino Island, Chile; 54°56′33.71″S, 67°37′42.36″W; hereafter referred to as Navarino); and 15 from Whalers’ Bay (Deception Island, Antarctica; 62°58′22.22″S, 60°34′32.55″W; hereafter referred to as Deception) (Fig. 1).

In Coyhaique, samples were collected from two *Nothofagus pumilio* forests; in Karukinka and Navarino, from *N. pumilio* forests and from grasslands without forest cover; and in Deception, from a volcanic hillside (see Table S1 in the Supporting Information for additional details).

In each sampling site, specimens that were phenotypically different, particularly in the color of the thalli, size of the lobes, presence or absence of reproductive structures and, if present, type of reproductive structures (sexual/asexual), were collected in order to sample most of the existing diversity of *Peltigera* at these locations. Thalli were sampled at least 1 m from the next closest thallus to avoid resampling the same genetic individual.

### Pre-treatment of samples and DNA extraction

The collected portions of the lichen thalli were superficially cleaned with a sterile brush and spatula, thoroughly rinsed with sterile distilled water, and air dried at room temperature. Eighty to 100 mg of each cleaned portion were mechanically fractioned with a mini-grinder, and DNA was extracted using the PowerSoil^TM^ DNA Isolation kit (MoBio Laboratories, Carlsbad, CA, USA) according to the manufacturer’s instructions. The quality and integrity of the extracted DNA were visualized in 0.8% (w/v) agarose gels in TAE 1× buffer (40 mM Tris-acetate, 1 mM EDTA [pH 8.0]) stained with GelRed^TM^ (Biotium, Hayward, CA, USA). All DNA samples were stored at −20°C until analysis.

### PCR amplification and sequencing

The fungal ITS region (ITS1-5.8S-ITS2) and LSU rDNA were amplified with primers ITS1 and ITS4 (57), and LIC24R (27) and LR7 (54), respectively.

Cyanobacterial SSU rDNA was amplified with the primers PCR1 and PCR18 (58).

The cycling conditions followed recommendations described in the studies above for each primer set using the GoTaq^®^ Green Master Mix (GoTaq^®^ DNA polymerase in 1× Green GoTaq^®^ Reaction Buffer [pH 8.5], 200 μM of each dNTP, and 1.5 mM MgCl_2_) (Promega, Madison, WI, USA) with a Maxygene thermocycler (Axygen, CA, USA). The quality and size of the amplicons were determined electrophoretically as described above, except that 1.2% (w/v) agarose gels were used.

All amplicons were sequenced in one direction with the forward primers using a sequencing service (Macrogen, Seoul, South Korea) in the Genetic Analyzer 3730XL (Applied Biosystems, Carlsbad, CA, USA).

### Sequence analyses

DNA sequences were visually checked and manually edited on Mega 5.2 software (52) and aligned with the Muscle alignment tool (8) provided in the same software. Edited sequence fragments from mycobionts and cyanobionts were both subjected to blast-n queries (1) for an initial verification of their identities in comparison to the non-redundant nucleotide database at GenBank (NCBI).

### Phylogenetic analyses

#### Mycobionts

In spite of extensive efforts to amplify the nuclear ribosomal internal transcribed spacer (ITS), it was not possible to obtain the high quality sequences necessary for a phylogenetic analysis in most cases (data not shown) and, thus, LSU rDNA was instead used to determine the phylogeny of the mycobiont.

Mycobiont operational taxonomic units (OTUs) were defined as the groups of sequences that were 100% identical (nucleotide identity), namely, no different sequences were included in the same OTU.

Phylogenetic analyses were performed on a fungal LSU rDNA sequence set consisting of one representative of each of the different mycobiont OTUs (*i.e.*, no repeated sequences were included) plus a selection of 67 *Peltigera* accessions selected from previous studies on this genus (14, 27, 28) and downloaded from GenBank. The accessions included one representative of the sequenced species reported in the above-mentioned studies (not including the Hydrotheriae clade; 30) and, if the same species was reported in more than a single geographic site, representatives of each site were included in the selection. The presence of ambiguously aligned nucleotides was determined using default parameters on the web server Guidance (38), and these were removed prior to subsequent phylogenetic reconstructions of maximum likelihood (ML) and Bayesian inference (BI). ML analyses were performed on the T-REX web server (5) under the PhyML algorithm. The best nucleotide substitution model was determined with the help of jModelTest 2.1.1. (39) under the corrected Akaike Information Criteria (AICc), which suggested the TIM2+I+G as the best fitting model of evolution for the ML analyses. Since this model could not be implemented in PhyML, the data set was analyzed using the GTR+I+G model of evolution because it was the closest model available in PhyML.

Bayesian Inference (BI) was carried out based on the same model of evolution selected and using the Metropolis-coupled Bayesian Markov chain Monte Carlo algorithm (MC)^3^ implemented in the software MrBayes 3.1.2 (16). Four independent runs were made of 10 million generations each, with the chains being sampled every 1,000 generations. The first 2,500 samples were discarded as burn-in, and convergence was assessed by examining all parameters using Tracer v. 1.5 (Rambaut *et al.* 2014 available at: http://beast.bio.ed.ac.uk/Tracer).

In the fungal analyses, *Solorina saccata* isolate AFTOL-ID 127 was used as an outgroup (accession number DQ973044). OTUs were named based on statistically supported nodes (Bootstrap >75% and PP >0.95) recovered in the phylogenetic analysis.

#### Cyanobionts

In the case of the cyanobionts, OTUs were defined under the same criteria used for the mycobionts, namely, each OTU was comprised of identical sequences. ML and BI phylogenetic reconstructions were performed on a cyanobacterial SSU rDNA sequence set that consisted of one representative of each cyanobacterial OTU plus 49 *Nostoc* SSU rDNA sequences downloaded from GenBank for comparisons. These sequences included a selection of the *Nostoc* sequences reported by O’Brien *et al.* (33), in addition to close matches to our cyanobionts according to the blast-n results if they were not already included in the selection from O’Brien *et al.* (33). Both phylogenetic analyses were performed as described in the case of the mycobionts, except that the TPM2uf+I+G was selected as the best nucleotide substitution model. This model could not be implemented in PhyML; therefore, the data set was analyzed using the GTR+I+G model of evolution as it was the most similar model available in PhyML. In the cyanobacterial analyses, *Fischerella muscicola* strain PCC7414 (accession number AF132788) was set as an outgroup.

All phylogenetic trees were drawn on the program TreeGraph 2.0.54-364 beta (51).

#### Rarefaction analyses

With the aim of determining whether the sampling was sufficient to cover the expected richness of the mycobionts and cyanobionts in each locality, the number of specimens in the different sampling sites was adjusted to a theoretical rarefaction curve by a non-linear regression (GraphPad Prism 4.0). In order to perform this adjustment, the values were first homogenized by extrapolating them to the number of specimens present at the sites in which the highest number of samples was collected (EstimateS; Colwell 2013 available at: http://purl.oclc.org/estimates). The coverage estimation was subsequently calculated according to *Cx = 1− (N_x_* */n)*, where *N_x_*: the number of operational taxonomic units (OTUs) and *n*: the total number of individuals. The Margalef index was then calculated according to *D_Mg_* *= (S−1)/ln N*, where *S*: the number of OTUs and *N*: the total number of individuals collected, and the Chao1 estimator according to *S_Chao1_* *= S_obs_+(n_1_^2^/2n_2_)*, where *S_obs_*: the number of observed OTUs, *n_1_*: the number of singletons (OTUs detected once), and *n_2_*: the number of doubletons (OTUs detected twice).

#### Nucleotide sequence accession numbers

The sequences obtained were deposited in the GenBank database under accession numbers KF718515 to KF718640 (LSU rDNA) and KF718389 to KF718514 (SSU rDNA), and were added to the dataset previously generated from Karukinka (41) (see Table S1 in the Supporting Information for additional details).

## Results

### Mycobionts

One hundred and eighty-six fungal LSU rDNA sequences were successfully amplified from the DNA extracted directly from the lichen samples. A blast-n analysis on these sequences confirmed that they all belonged to the genus *Peltigera*. Among the 186 amplicons, 8 different OTUs were established and named from M1 to M8 (see Table S1 in the Supporting Information for additional details). The morphological characteristics analyzed confirmed these groupings since morphology differed among them, but was consistent within each group.

**Fig. 2:**
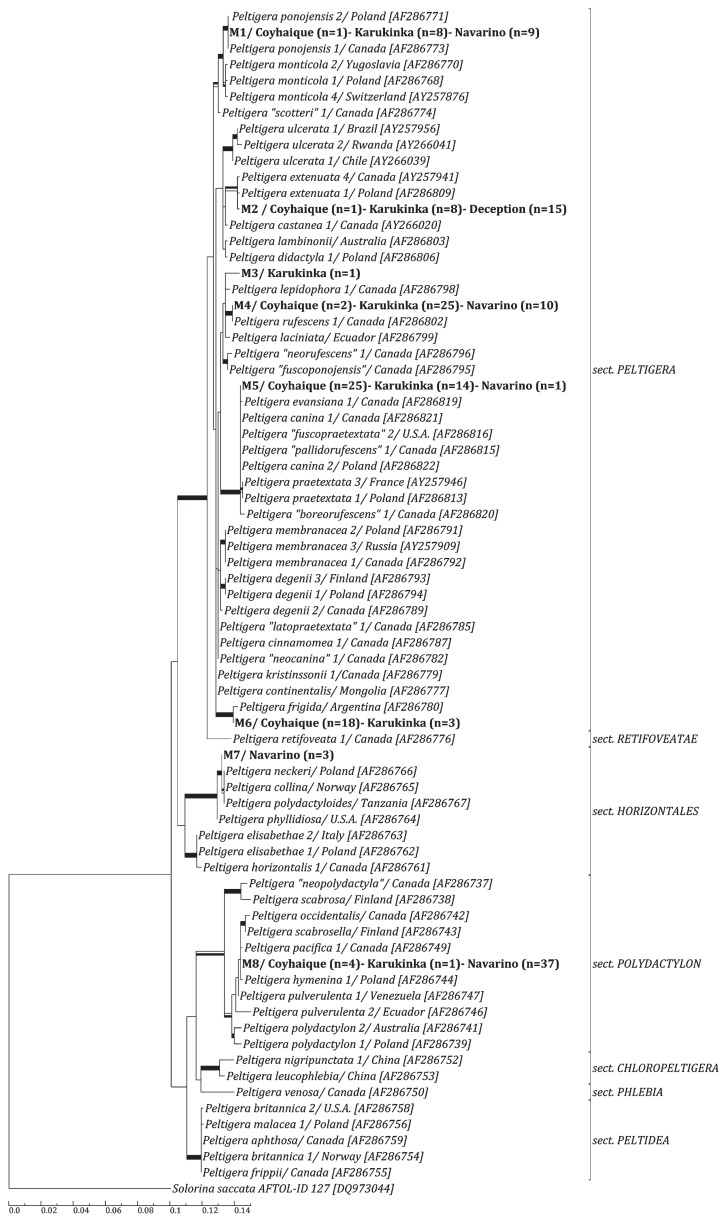
Phylogenetic relationships among *Peltigera* mycobionts. Phylogeny was based on a maximum likelihood analysis of 76 LSU rDNA sequences, which included 728 nucleotide positions. Support values are indicated as wide branches for nodes that received significant support by either maximum-likelihood bootstrap values ≥75% (black upper half), Bayesian Inference pp values ≥0.95 (black lower half), or both (complete black rectangle). The mycobionts from this study, named M1 to M8, are shown in bold. Their geographical origin is also indicated for each mycobiont and the number of specimens per sampling site is shown between parentheses. The division of the species into sections corresponds to the classification proposed by Miadlikowska & Lutzoni (27). Taxa in quotation marks correspond to those that have not yet been formally published. Codes in square brackets next to the sequences downloaded from the database correspond to their accession numbers. The geographical origin of these specimens is also shown next to their names and separated by a slash.

In order to determine the relationship of these specimens to other *Peltigera* lichens from the rest of the world, a sequence set was created consisting of one representative of each group plus the 67 LSU rDNA *Peltigera* sequences downloaded from GenBank, giving a final set of 76 sequences with 728 nucleotide positions each. This sequence set was subjected to ML and BI analyses and, given that both yielded similar tree topologies, only the best tree obtained from the ML was shown (Fig. 2). All parameters from the Bayesian analysis reached convergence across runs, with estimated sample sizes exceeding 200, potential scale reduction factors all equal to 1.00, and the average standard deviation of split frequencies equal to 0.004.

Phylogenetic reconstructions placed mycobionts M1 to M8 in different positions within *Peltigera* (Fig. 2, bold names). According to the classification of this genus into the sections proposed by Miadlikowska & Lutzoni (27), our samples belonged to sections *Polydactylon* (M8), *Horizontales* (M7), and *Peltigera* (M1 to M6).

Within these sections, most of our mycobionts formed defined and well-supported monophyletic groups (>75% bootstrap and >0.95 posterior probabilities) with some of the other *Peltigera* species downloaded from the database: M1 was closely related to *P. ponojensis*, M2 to *P. extenuata*, M4 to *P. rufescens*, and M6 to *P. frigida*. The rest of our mycobionts were closely related to more than one species, also forming defined and well-supported monophyletic lineages: M5 with *P. evansiana* / *P. canina* / *P. “fuscopraetextata”* / *P. “pallidorufescens”* / *P. praetextata* / *P. “boreorufescens” (*hereafter the *P. canina* lineage), M7 with *P. neckeri* / *P. collina* / *P. polydactyloides* (hereafter the *P. neckeri* lineage), and M8 with *P. polydactylon* / *P. occidentalis* / *P. scabrosella*/ *P. pacifica* / *P. hymenina* / *P. pulverulenta* (hereafter the *P. hymenina* lineage). M3 was the only mycobiont that was not closely related to any of the sequences downloaded from the database and, thus, was not classified beneath the section level. In spite of several attempts to obtain high quality ITS sequences for this sample, it was only possible for a 200 nucleotide fragment of this region. A blast-n analysis of this fragment showed, with a 98% nucleotide identity, that this specimen may be related to *P. “granulosa”* and *P. “papuana”*, both described by Sérusiaux *et al.* (47) as new species from New Guinea. Moreover, its ITS1-HR (ITS hyper-variable region) (28) was similar to that proposed for the new putative group *P. “papuanorum”*, which includes both species and was briefly described by Lutzoni *et al.* (available at: http://www.peltigera.lutzonilab.net). However, the ITS1-HR of M3 was interrupted by an insert of 86 nucleotides, which suggested that it may belong to a new, still undescribed, species.

According to the rarefaction curves, the theoretically expected mycobiont types at each sampling site were 7, 8, 5, and 1 for Coyhaique, Karukinka, Navarino, and Deception, respectively, which were consistent with the values predicted by the Margalef index and Chao1 estimator (Table 1). Therefore, the sampling covered 88%, 88%, 92%, and 93%, of the expected *Peltigera* species richness at Coyhaique, Karukinka, Navarino, and Deception, respectively (Table 1).

### Cyanobionts

All 186 cyanobacterial SSU rDNA sequences were successfully amplified from the lichen samples. Every sequencing reaction produced clean reads with no secondary peaks, suggesting that only a single photobiont genotype dominated in each lichen thallus. A blast-n analysis on these sequences confirmed that they all belonged to the genus *Nostoc*. Among the 186 amplicons, 15 different OTUs were established and named from C1 to C15 (see Table S1 in the Supporting Information for additional details).

**Fig. 3:**
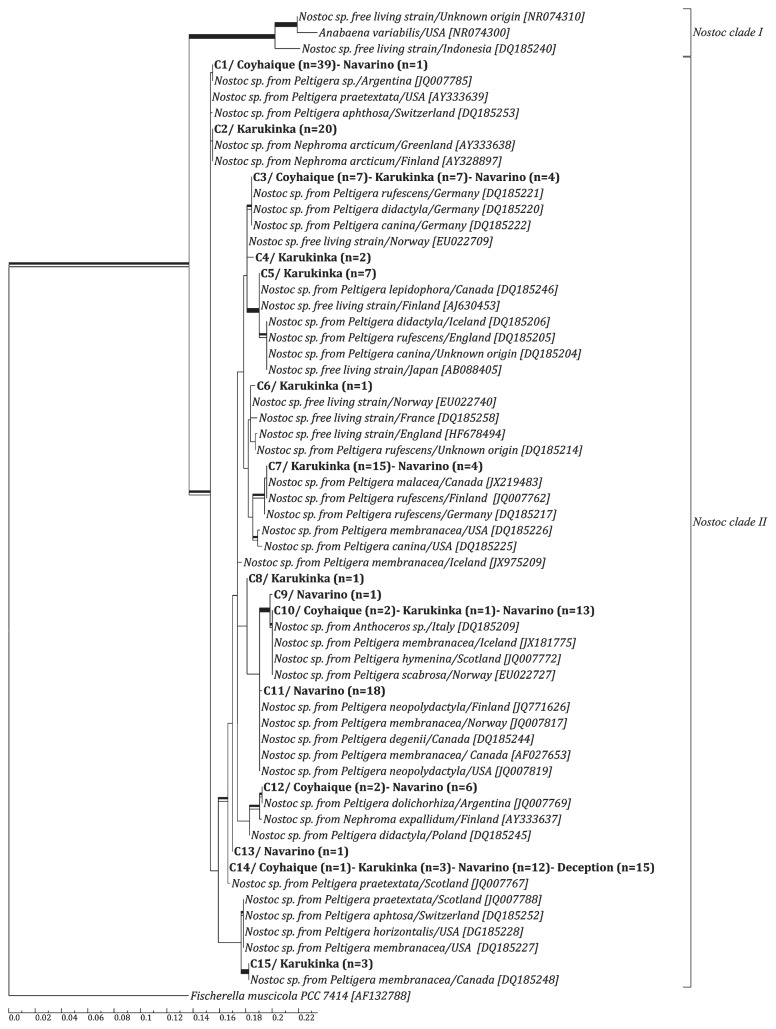
Phylogenetic relationships among *Nostoc* cyanobionts. Phylogeny was based on maximum likelihood analyses of 65 SSU rDNA sequences, which included 690 nucleotide positions. Support values are indicated as wide branches for nodes that received significant support by either maximum-likelihood bootstrap values ≥75% (black upper half), Bayesian Inference pp values ≥0.95 (black lower half), or both (complete black rectangle). The cyanobionts from this study, named C1 to C15, are shown in bold. Their geographical origin is also indicated and the number of specimens per sampling site is shown between parentheses. The division of the species into *Nostoc* clades I and II corresponds to the classification proposed by O’Brien *et al.* (33). Codes in square brackets next to the sequences downloaded from the database correspond to their accession numbers. The geographical origin of these specimens is also shown next to their names and separated by a slash.

In order to determine how these cyanobionts were related to other *Nostoc* strains from different parts of the world, a sequence set was created, consisting of one representative of each of our 15 groups along with the 49 *Nostoc* SSU rDNA sequences downloaded from the GenBank database, giving a final set of 65 sequences with 690 nucleotide positions each. Phylogenetic analyses of ML and BI were performed on this sequence set and, since both yielded similar tree topologies, only the best tree obtained from the ML is shown (Fig. 3). All parameters from the Bayesian analysis reached convergence across runs, with estimated sample sizes exceeding 200, potential scale reduction factors all equal to 1.00, and average standard deviation of split frequencies equal to 0.006.

The analyses showed that all our cyanobionts belonged to the *Nostoc* II clade proposed by O’Brien *et al.* (33) (Fig. 3), and the *Peltigera* guild proposed by Rikkinen *et al.* (43) (data not shown).

All the *Nostoc* sequences reported in this study were related (>97.5% nucleotide identity) to cyanobionts previously found in other lichens such as *Peltigera* and *Nephroma* from North America and Europe, except C6, which was more related to European free living *Nostoc* strains, and C1 and C12, more closely-related to cyanobionts described from South American *Peltigera* specimens.

According to the rarefaction analysis, we expected 6, 12, 10, and 1 cyanobiont types at each sampling site, for Coyhaique, Karukinka, Navarino, and Deception, respectively. For their part, the Margalef index and the Chao1 estimator resulted in similar richness values (Table 1) and, hence, the coverage of the expected cyanobiont richness reached by the sampling was of 90%, 83%, 85%, and 93%, for Coyhaique, Karukinka, Navarino, and Deception, respectively (Table 1).

## Discussion

The rarefaction analyses, which were carried out to estimate the representativeness of the sampling, showed, in the case of the mycobionts, that it was sufficient to cover the vast majority of the theoretical richness of *Peltigera* species in the different locations, reaching approximately 90% of the expected richness values. In the case of the cyanobionts, the sampling covered over 80% of the theoretical richness of *Nostoc* in these locations. Therefore, our sampling approach, which was not based on *a priori* species hypotheses, captured a large amount of the *Peltigera* and *Nostoc* diversities in the sites examined, offering an effective way to gain an insight into the diversity of the species and their interactions in poorly-studied locations.

### Mycobionts

By intensively sampling terricolous *Peltigera* spp. across four sites in Southern Chile and Antarctica, we detected 8 different mycobionts and 15 cyanobionts in the 186 samples tested, which represented a high proportion of the expected diversity. A phylogenetic reconstruction of the mycobionts, including sequences from Europe, North America, Asia, and Australia and a few from South America (Argentina, Brazil, Chile, Ecuador, and Venezuela), which were all previously described in key treatments (*e.g.* 14, 27, 28), showed that most of our mycobionts (6/8) were part of the infra-generic section *Peltigera*, while the other 2 belonged to the sections *Horizontales* and *Polydactylon*, respectively (27). Four out of the 8 OTUs (M1, M2, M4, and M6) were placed with a relatively high degree of confidence, supported on short branches, within clusters comprised of single-species accessions (Fig. 2).

Few studies have investigated the diversity of *Peltigera* in the regions examined in the present study; being the general revision of the distribution patterns in the genus performed by Martínez *et al.* (25), the only study that covered the four sampling sites. Quilhot *et al.* (40) reported the lichen diversity in Aysén Region (~110,000 km^2^), in which the Coyhaique National Reserve (21,500 km^2^) is located, and detected 13 species from which one of our mycobionts was not reported (M1, related to *P. ponojensis*). On the other hand, the only published studies on *Peltigera* at Karukinka corresponded to a previous baseline study conducted in different areas of the park (2) and our previous studies (41, 42), which included the same Karukinka specimens analyzed in the present study. In the other two sites, previous studies only found a small number of species including *P. rufescens* in the case of Navarino (26) and *P. didactyla* in Deception Island (3, 37, 49). In this last case, the only mycobiont present in Antarctica (M2) was strongly related to *P. extenuata*, previously included in the *P. didactyla* complex (14). Given that the species belonging to this complex displayed similar morphological characteristics, we are unable to discern which of them correspond to the ones previously reported in Deception. Not all the species previously identified based on morphological characteristics (40) were detected in this study; these species may have been present at the sites, but were rare enough to remain un-sampled. However, the present study identified the presence of other OTUs not previously reported in the study areas, suggesting that the diversity of *Peltigera* was underestimated in these locations.

The distribution of the lineages documented in this study fit some biogeographic expectations, such as the tripartite *Peltigera* species not being detected at the sampled locations, regardless of their apparent abundance in studies from the northern hemisphere (34, 56), and also *P. frigida* (related to M6) being exclusively described in the southernmost part of South America (25, 27). However, some of our results were unexpected. For example, species from the *P. neckeri* lineage (related to M7) and *P. hymenina* lineage (related to M8) were rare in South America (25, 40), being defined commonly as circumpolar (25). Furthermore, to the best of our knowledge, there have been no reports of *P. ponojensis* (related to M1) in South America, except for our previous studies from Karukinka (41, 42). One specimen, M3, was not related to any published sequence from the database, indicating that it could corresponded to a new undescribed species. Since *Peltigera* has been poorly explored in the sampled regions, it is possible that this lichen corresponds to a novel South American species; however, we cannot rule out the possibility that a DNA-based identification, despite its great potential to accurately identify a high percentage of specimens to the correct species, is limited by the availability of accurate baseline taxonomic data (4, 32, 36). Moreover, apart from not being accurately identified, this mycobiont may correspond to a rare species because, from the nearly 200 lichens sampled, it was only represented by a single specimen.

In spite of extensive efforts to amplify the nuclear ribosomal internal transcribed spacer (ITS), which has been proposed as the universal DNA barcode marker for Fungi (46), it was not possible to obtain high quality sequences in many cases. A possible explanation is that mononucleotide runs in the ITS, which are known to cause problems with Sanger sequencing (18), are common in the genus *Peltigera* (28). Although these problems can be overcome in most cases by sequencing in both directions, this was not achievable in the scope of this study and, thus, LSU rDNA was used instead, even though it is not the highest resolution marker available for fungi. Therefore, it is worth considering these results in the possible light of the further resolution of mycobionts.

Even though we were relatively confident about the identity of half of our recognized specimens due to their short branch lengths and strong statistical support in the phylogenetic analyses, identification using comparisons with extant sequenced vouchers opened two possibilities that must be considered. Sequenced voucher specimens may be misidentified, which is not uncommon, particularly for fungal accessions in GenBank (6, 32). Furthermore, unrecognized diversity may exist for the relatively poorly explored South American continent (25); therefore, identified or sequenced vouchers do not exist for some of the lineages actually present in Chile. Further studies are required to fully explore the morphological and genetic diversity present using more surveys in addition to higher resolution markers.

### Cyanobionts

Regarding the cyanobionts, all symbiotic *Nostoc* sequences determined in this study fell within the *Peltigera* guild reported by Rikkinen *et al.* (43) and within the *Nostoc* II clade reported by O’Brien *et al.* (33). According to these studies, all *Nostoc* strains that are symbiotically associated with *Peltigera* belong to this group, along with other symbiotic and free living strains, and they may all comprise one single species.

The sequences related to the cyanobionts of the present study were generally from specimens collected in North America and Europe, with the exceptions of C1 and C12, which were the most similar to cyanobionts previously described from South America, particularly from Argentina (17), and C4, C8, and C13, which did not closely associate with any known *Nostoc* species. C8 is of particular interest because it was exclusively associated with M3, the mycobiont that did not relate with any of the known *Peltigera*. None of the cyanobacterial sequences of the present study was closely related to sequences from Oceania, also from *Nostoc* clade II, despite the recognized floristic relationship, for example, between New Zealand and Southern Chile (9). The only cyanobiont present in Antarctica, C14, was closely related to a *Peltigera* cyanobiont from Scotland (17), a humid and temperate region with conditions very distant from those present in Antarctica. However, it was also present in lichens from Coyhaique, Karukinka, and Navarino, which suggests this is a versatile strain capable of adapting to different ecological conditions. In conclusion, the *Nostoc* sequences from the present study did not present strong geographic patterns, which was consistent with previous findings published by Stenroos *et al.* (50) and Wirtz *et al.* (59) whose *Nostoc* clades did not correlate with the geographic origin of the lichens, suggesting that some *Nostoc* taxa are widely distributed.

## Supplementary Information



## Figures and Tables

**Table 1:** Rarefaction, estimated richness coverage and diversity parameters for mycobionts and cyanobionts from different sampling sites.

	Site	Observed types	Sampled specimens	Theoretical types^a^	R^2^	Coverage (%)	Margalef index	Chao1 estimator
Mycobionts	Coyhaique	6	51	7	0.9674	88	5.7	6.5
	Karukinka	7	60	8	0.9899	88	6.8	8.0
	Navarino	5	60	5	0.9904	92	4.8	5.0
	Deception	1	15	1	ND	93	0.6	1.0

Cyanobionts	Coyhaique	5	51	6	0.9804	90	4.8	5.0
	Karukinka	10	60	12	0.9941	83	9.8	11.5
	Navarino	9	60	10	0.9932	85	8.8	12.0
	Deception	1	15	1	ND	93	0.6	1.0

a The theoretical number of OTUs in each sampling site was inferred by adjusting each rarefaction curve to a theoretical curve by non-linear regression (see *Materials and Methods* for details).

## References

[1] Altschul SF, Gish W, Mille W, Myers EW, Lipman DJ (1990). Basic local alignment search tool. J Mol Biol.

[2] Arroyo M, Donoso C, Murúa R, Pisano E, Schlatter R, Serey I (1996). Towards an ecologically sustainable forestry project: concepts, analyses and recommendations (originally in Spanish).

[3] ASPA 140 (2005). Management Plan for Antarctic Specially Protected Area No. 140: Parts of Deception Island, South Shetland Islands.

[4] Begerow D, Nilsson H, Unterseher M, Maier W (2010). Current state and perspectives of fungal DNA barcoding and rapid identification procedures. Appl Microbiol Biotechnol.

[5] Boc A, Diallo AB, Makarenkov V (2012). T-REX: a web server for inferring, validating and visualizing phylogenetic trees and networks. Nucleic Acids Res.

[6] Bridge PD, Roberts PJ, Spooner BM, Panchal G (2003). On the unreliability of published DNA sequences. New Phytol.

[7] Crespo A, Lumbsch HT (2010). Cryptic species in lichen-forming fungi. IMA Fungus.

[8] Edgar RC (2004). MUSCLE: multiple sequence alignment with high accuracy and high throughput. Nucleic Acids Res.

[9] Ezcurra C, Baccalá N, Wardle P (2008). Floristic relationships among vegetation types of New Zealand and the Southern Andes: similarities and biogeographic implications. Ann Bot.

[10] Friedl T, Büdel B, Nash TH (2008). Photobionts. Lichen Biology.

[11] Galloway DJ (2000). The lichen genus *Peltigera* (Peltigerales: Ascomycota) in New Zealand. Tubinga.

[12] Galloway DJ (2010). Flora of New Zealand: Lichens, including lichen-forming and lichenicolous fungi. Folia Geobotanica.

[13] Goffinet B, Hastings RI (1994). The lichen genus *Peltigera (* Lichenized Ascomycetes) in Alberta. Natural History Occasional Paper 21.

[14] Goffinet B, Miadlikowska J, Goward T (2003). Phylogenetic inferences based on nrDNA sequences support five morphospecies within the *Peltigera didactyla* complex (lichenized Ascomycota). Bryologist.

[15] Goward T, Goffinet B, Vitikainen O (1995). Synopsis of the genus *Peltigera* (lichenized Ascomycetes) in British Columbia, with a key to the North American species. Can J Bot.

[16] Huelsenbeck JP, Ronquist F (2001). MRBAYES: Bayesian inference of phylogeny. Bioinformatics.

[17] Kaasalainen U, Fewer DP, Jokela J, Wahlsten M, Sivonen K, Rikkinen J (2012). Cyanobacteria produce a high variety of hepatotoxic peptides in lichen symbiosis. Proc Natl Acad Sci USA.

[18] Kircher M, Kelso J (2010). High-throughput DNA sequencing— concepts and limitations. BioEssays.

[19] Kirk PM, Cannon PF, Minter DW, Stalpers JA (2008). Dictionary of the Fungi.

[20] Kroken S, Taylor JW (2000). Phylogenetic species, reproductive mode, and specificity of the green alga *Trebouxia* forming lichens with the fungal genus *Letharia*. Bryologist.

[21] Louwhoff S (2008). New and additional records and a new combination of Australian *Peltigera*, Australas. Lichenologist.

[22] Lücking R, Lawrey JD, Sikaroodi M, Gillevet PM, Chaves JL, Sipman HJ, Bungartz F (2009). Do lichens domesticate photobionts like farmers domesticate crops? Evidence from a previously unrecognized lineage of filamentous cyanobacteria. Am J Bot.

[23] Lücking R, Dal-Forno M, Sikaroodi M, Gillevet PM, Bungartz F, Moncada B, Yánez-Ayabacac A, Chavese JL, Coca LF, Lawrey JD (2014). A single macrolichen constitutes hundreds of unrecognized species. Proc Natl Acad Sci USA.

[24] Lumbsch HT, Leavitt SD (2011). Goodbye morphology? A paradigm shift in the delimitation of species in lichenized fungi. Fungal Divers.

[25] Martínez I, Burgaz A, Vitikainen O, Escudero A (2003). Distribution patterns in the genus *Peltigera* Willd. Lichenologist.

[26] Méndez M, Rozzi R, Cavieres L, Goffinet B, Rozzi R, Lewis L, Buck W, Massardo F (2012). Moss and lichen on the subantarctic Andean gardens (Spanish). The Miniature Forests of Cape Horn: Eco-tourism with a hand-lens.

[27] Miadlikowska J, Lutzoni F (2000). Phylogenetic revision of the genus *Peltigera* (lichen-forming Ascomycota) based on morphological, chemical, and large subunit nuclear ribosomal DNA data. Int J Plant Sci.

[28] Miadlikowska J, Lutzoni F, Goward T, Zoller S, Posada D (2003). New approach to an old problem: Incorporating signal from gap-rich regions of ITS and rDNA large subunit into phylogenetic analyses to resolve the *Peltigera canina* species complex. Mycologia.

[29] Miadlikowska J, Lutzoni F (2004). Phylogenetic classification of peltigeralean fungi (Peltigerales, Ascomycota) based on ribosomal RNA small and large subunits. Am J Bot.

[30] Miadlikowska J, Richardson DM, Magain N, Ball B, Anderson F, Cameron R, Lendemer J, Truong C, Lutzoni F (2014). Phylogenetic placement, species delimitation, and cyanobiont identity of endangered aquatic *Peltigera* species (lichen-forming Ascomycota, Lecanoromycetes). Am J Bot.

[31] Nelsen MP, Rivas-Plata E, Andrew CJ, Lücking R, Lumbsch HT (2011). Phylogenetic diversity of trentepohlialean algae associated with lichen-forming fungi. J Phycol.

[32] Nilsson RH, Ryberg M, Kristiansson E, Abarenkov K, Larsson KH, Kõljalg U (2006). Taxonomic reliability of DNA sequences in public sequence databases: A fungal perspective. PLoS ONE.

[33] O’Brien HE, Miadlikowska J, Lutzoni F (2005). Assessing host specialization in symbiotic cyanobacteria associated with four closely related species of the lichen fungus *Peltigera*. Eur J Phycol.

[34] O’Brien HE, Miadlikowska J, Lutzoni F (2009). Assessing reproductive isolation in highly diverse communities of the lichen-forming fungal genus *Peltigera*. Evolution.

[35] O’Brien H (2013). Green Algal Photobionts: Trebouxia.

[36] Orock EA, Leavitt SD, Fonge BA, St Clair LL, Lumbsch HT (2012). DNA-based identification of lichen-forming fungi: can publicly available sequence databases aid in lichen diversity inventories of Mount Cameroon (West Africa)?. Lichenologist.

[37] Øvstedal DO, Lewis-Smith RI (2001). Lichens of Antarctica and South Georgia. A Guide to Their Identification and Ecology.

[38] Penn O, Privman E, Ashkenazy H, Landan G, Graur D, Pupko T (2010). GUIDANCE: a web server for assessing alignment confidence scores. Nucleic Acids Res.

[39] Posada D (2008). jModelTest: Phylogenetic Model Averaging. Mol Biol Evol.

[40] Quilhot W, Cuellar M, Diaz R, Riquelme F, Rubio C (2012). Lichens of Aisen, Southern Chile. Gayana Bot.

[41] Ramírez-Fernández L, Zúñiga C, Méndez M, Carú M, Orlando J (2013). Genetic diversity of terricolous *Peltigera* cyano-lichens communities in different conservation states of native forest from Southern Chile. Int Microbiol.

[42] Ramírez-Fernández L, Zúñiga C, Carú M, Orlando J (2014). Environmental context shapes the bacterial community structure associated to *Peltigera* cyanolichens growing in Tierra del Fuego, Chile. World J Microbiol Biot.

[43] Rikkinen J, Oksanen I, Lohtander K (2002). Lichen guilds share related cyanobacterial symbionts. Science.

[44] Rikkinen J (2013). Molecular studies on cyanobacterial diversity in lichen symbioses. MycoKeys.

[45] Sadowska-Des AD, Dal Grande F, Lumbsch HT, Beck A, Otte J, Hur JS, Kim JA, Schmitt I (2014). Integrating coalescent and phylogenetic approaches to delimit species in the lichen photobiont *Trebouxia*. Mol Phylogenet Evol.

[46] Schoch CL, Seifert KA, Huhndorf S, Robert V, Spouge JL, Levesque CA, Chen W, Fungal Barcoding Consortium (2012). Nuclear ribosomal internal transcribed spacer (ITS) region as a universal DNA barcode marker for Fungi. Proc Natl Acad Sci USA.

[47] Sérusiaux E, Goffinet B, Miadlikowska J, Vitikainen O (2009). Taxonomy, phylogeny and biogeography of the lichen genus *Peltigera* in Papua New Guinea. Fungal Div.

[48] Škaloud P, Peksa O (2010). Evolutionary inferences based on ITS rDNA and actin sequences reveal extensive diversity of the common lichen alga *Asterochloris* (Trebouxiophyceae, Chlorophyta). Mol Phylogenet Evol.

[49] Spielmann AA, Pereira AB (2012). Lichens on the maritime Antarctica (A small field guide for some common species). Glalia.

[50] Stenroos S, Högnabba F, Myllys L, Hyvönen J, Thell A (2006). High selectivity in symbiotic associations of lichenized ascomycetes and cyanobacteria. Cladistics.

[51] Stöver BC, Müller KF (2010). TreeGraph 2: Combining and visualizing evidence from different phylogenetic analyses. BMC Bioinformatics.

[52] Tamura K, Peterson D, Peterson N, Stecher G, Nei M, Kumar S (2011). MEGA5: molecular evolutionary genetics analysis using maximum likelihood, evolutionary distance, and maximum parsimony methods. Mol Biol Evol.

[53] Thüs H, Muggia L, Pérez-Ortega S (2011). Revisiting photobiont diversity in the lichen family Verrucariaceae (Ascomycota). Eur J Phycol.

[54] Vilgalys R, Hester M (1990). Rapid genetic identification and mapping of enzymatically amplified ribosomal DNA from several *Cryptococcus* species. J Bacteriol.

[55] Vitikainen O (2004). Two New Zealand species of *Peltigera* revisited. Symb Bot Ups.

[56] Wei XL, Wang XY, Koh YJ, Hur JS (2009). Taxonomic study of *Peltigera* (Peltigeraceae, Ascomycota) in Korea. Mycobiology.

[57] White TJ, Bruns T, Lee S, Taylor J, Innis MA, Gelfand DH, Sninsky JJ, White TJ (1990). Amplification and direct sequencing of fungal ribosomal RNA genes for phylogenetics. PCR Protocols: a Guide to Methods and Applications.

[58] Wilmotte A, Van der Auwera G, De Wachter R (1993). Structure of the 16S ribosomal RNA of the thermophilic cyanobacterium *Chlorogloeopsis* HTF (‘*Mastigocladus laminosus* HTF’) strain PCC7518, and phylogenetic analysis. FEBS Lett.

[59] Wirtz N, Lumbsch HT, Green TG, Türk R, Pintado A, Sancho L, Schroeter B (2003). Lichen fungi have low cyanobiont selectivity in maritime Antarctica. New Phytologist.

