# Supplementary material for: Phylogenetic Diversity of Peltigera Cyanolichens and Their Photobionts in Southern Chile and Antarctica
Source: Microbes Environ. 2015 Apr 28;30(2):172–9. doi: 10.1264/jsme2.ME14156 (PMC4462928; doi:10.1264/jsme2.ME14156)
Supplement: Supplementary file 1 [file 30_172_s1.pdf]

## Supporting Information

**Table S1.** Sampling sites, samples and sequences information.

| Sampling site information | | | | | Samples information (geo-reference data) | | | Sequences information (accession numbers) | |
|---|---|---|---|---|---|---|---|---|---|
| Site | Environmental Context | Sample | *Ms* OTU | *Cs* OTU | Latitude (S) | Longitude (W) | Altitude | 28S rRNA gene* | 16S rRNA gene* |
| Coyhaique | *N. pumilio* middle-aged forest 1 | C1 | M5 | C1 | -45,527495 | -72,033067 | 654,8 m | KF718515 | KF718389 |
| | *N. pumilio* middle-aged forest 1 | C2 | M5 | C1 | -45,527590 | -72,032775 | 688,6 m | KF718516 | KF718390 |
| | *N. pumilio* middle-aged forest 1 | C3 | M5 | C1 | -45,527590 | -72,032817 | 688,5 m | KF718517 | KF718391 |
| | *N. pumilio* middle-aged forest 1 | C4 | M8 | C12 | -45,527640 | -72,032805 | 696,4 m | KF718518 | KF718392 |
| | *N. pumilio* middle-aged forest 1 | C5 | M6 | C1 | -45,527840 | -72,032802 | 717,6 m | KF718519 | KF718393 |
| | *N. pumilio* middle-aged forest 1 | C6 | M6 | C1 | -45,527687 | -72,033257 | 709,8 m | KF718520 | KF718394 |
| | *N. pumilio* middle-aged forest 1 | C8 | M6 | C1 | -45,527767 | -72,033582 | 725,6 m | KF718521 | KF718395 |
| | *N. pumilio* middle-aged forest 1 | C9 | M6 | C1 | -45,527697 | -72,033745 | 712,4 m | KF718522 | KF718396 |
| | *N. pumilio* middle-aged forest 1 | C10 | M6 | C1 | -45,527677 | -72,033752 | 708,5 m | KF718523 | KF718397 |
| | *N. pumilio* middle-aged forest 1 | C11 | M6 | C1 | -45,527770 | -72,033735 | 713,4 m | KF718524 | KF718398 |
| | *N. pumilio* middle-aged forest 1 | C12 | M6 | C1 | -45,527662 | -72,033667 | 679,0 m | KF718525 | KF718399 |
| | *N. pumilio* middle-aged forest 1 | C13 | M6 | C1 | -45,527695 | -72,034010 | 717,4 m | KF718526 | KF718400 |
| | *N. pumilio* middle-aged forest 1 | C14 | M5 | C1 | -45,527637 | -72,034177 | 712,7 m | KF718527 | KF718401 |
| | *N. pumilio* middle-aged forest 1 | C15 | M5 | C1 | -45,527640 | -72,034165 | 710,8 m | KF718528 | KF718402 |
| | *N. pumilio* middle-aged forest 1 | C16 | M5 | C1 | -45,527640 | -72,034220 | 711,3 m | KF718529 | KF718403 |
| | *N. pumilio* middle-aged forest 1 | C17 | M5 | C1 | -45,527675 | -72,034190 | 713,0 m | KF718530 | KF718404 |
| | *N. pumilio* middle-aged forest 1 | C18 | M6 | C1 | -45,527462 | -72,034410 | 724,1 m | KF718531 | KF718405 |
| | *N. pumilio* middle-aged forest 1 | C19 | M6 | C1 | -45,527435 | -72,034137 | 724,2 m | KF718532 | KF718406 |
| | *N. pumilio* middle-aged forest 1 | C20 | M6 | C1 | -45,527682 | -72,034802 | 756,6 m | KF718533 | KF718407 |
| | *N. pumilio* middle-aged forest 1 | C21 | M8 | C10 | -45,527642 | -72,034835 | 729,8 m | KF718534 | KF718408 |
| | *N. pumilio* middle-aged forest 1 | C22 | M5 | C1 | -45,527555 | -72,035065 | 694,3 m | KF718535 | KF718409 |
| | *N. pumilio* middle-aged forest 1 | C23 | M5 | C1 | -45,527477 | -72,035210 | 697,2 m | KF718536 | KF718410 |
| | *N. pumilio* middle-aged forest 1 | C24 | M5 | C1 | -45,527652 | -72,035597 | 727,1 m | KF718537 | KF718411 |
| | *N. pumilio* middle-aged forest 1 | C25 | M5 | C14 | -45,527497 | -72,035997 | 709,8 m | KF718538 | KF718412 |
| | *N. pumilio* middle-aged forest 1 | C26 | M8 | C10 | -45,527588 | -72,036150 | 695,9 m | KF718539 | KF718413 |
| | *N. pumilio* middle-aged forest 1 | C27 | M6 | C1 | -45,527346 | -72,036335 | 720,7 m | KF718540 | KF718414 |
| | *N. pumilio* middle-aged forest 2 | C28 | M5 | C1 | -45,530000 | -72,026762 | 705,4 m | KF718541 | KF718415 |
| | *N. pumilio* middle-aged forest 2 | C29 | M6 | C1 | -45,529782 | -72,026792 | 713,9 m | KF718542 | KF718416 |
| | *N. pumilio* middle-aged forest 2 | C30 | M5 | C1 | -45,529692 | -72,026937 | 708,9 m | KF718543 | KF718417 |

| Sampling site information | | | | | Samples information (geo-reference data) | | | Sequences information (accession numbers) | |
|---|---|---|---|---|---|---|---|---|---|
| **Site** | **Environmental Context** | **Sample** | ***Ms* OTU** | ***Cs* OTU** | **Latitude (S)** | **Longitude (W)** | **Altitude** | **28S rRNA gene[*]** | **16S rRNA gene[*]** |
| | *N. pumilio* middle-aged forest 2 | C31 | M5 | C1 | -45,529690 | -72,026915 | 708,9 m | KF718544 | KF718418 |
| | *N. pumilio* middle-aged forest 2 | C32 | M4 | C3 | -45,529627 | -72,027347 | 704,6 m | KF718545 | KF718419 |
| | *N. pumilio* middle-aged forest 2 | C33 | M1 | C3 | -45,529802 | -72,027360 | 704,5 m | KF718546 | KF718420 |
| | *N. pumilio* middle-aged forest 2 | C34 | M5 | C3 | -45,529910 | -72,027467 | 705,1 m | KF718547 | KF718421 |
| | *N. pumilio* middle-aged forest 2 | C35 | M5 | C3 | -45,529882 | -72,027362 | 706,6 m | KF718548 | KF718422 |
| | *N. pumilio* middle-aged forest 2 | C36 | M5 | C1 | -45,529832 | -72,027367 | 704,4 m | KF718549 | KF718423 |
| | *N. pumilio* middle-aged forest 2 | C37 | M5 | C3 | -45,529760 | -72,027592 | 691,0 m | KF718550 | KF718424 |
| | *N. pumilio* middle-aged forest 2 | C38 | M6 | C1 | -45,529610 | -72,027377 | 708,5 m | KF718551 | KF718425 |
| | *N. pumilio* middle-aged forest 2 | C39 | M5 | C1 | -45,529490 | -72,027647 | 707,0 m | KF718552 | KF718426 |
| | *N. pumilio* middle-aged forest 2 | C40 | M5 | C1 | -45,529450 | -72,027647 | 710,8 m | KF718553 | KF718427 |
| | *N. pumilio* middle-aged forest 2 | C41 | M5 | C1 | -45,529520 | -72,027602 | 710,4 m | KF718554 | KF718428 |
| | *N. pumilio* middle-aged forest 2 | C42 | M5 | C1 | -45,529527 | -72,027627 | 703,8 m | KF718555 | KF718429 |
| | *N. pumilio* middle-aged forest 2 | C43 | M2 | C3 | -45,529455 | -72,027792 | 702,1 m | KF718556 | KF718430 |
| | *N. pumilio* middle-aged forest 2 | C44 | M4 | C3 | -45,529595 | -72,027832 | 702,8 m | KF718557 | KF718431 |
| | *N. pumilio* middle-aged forest 2 | C45 | M5 | C1 | -45,529515 | -72,027832 | 710,2 m | KF718558 | KF718432 |
| | *N. pumilio* middle-aged forest 2 | C46 | M5 | C1 | -45,529797 | -72,028022 | 712,0 m | KF718559 | KF718433 |
| | *N. pumilio* middle-aged forest 2 | C47 | M6 | C1 | -45,529702 | -72,028032 | 705,0 m | KF718560 | KF718434 |
| | *N. pumilio* middle-aged forest 2 | C48 | M6 | C1 | -45,529637 | -72,028160 | 702,3 m | KF718561 | KF718435 |
| | *N. pumilio* middle-aged forest 2 | C49 | M5 | C1 | -45,529600 | -72,028220 | 702,7 m | KF718562 | KF718436 |
| | *N. pumilio* middle-aged forest 2 | C50 | M6 | C1 | -45,529447 | -72,028465 | 703,0 m | KF718563 | KF718437 |
| | *N. pumilio* middle-aged forest 2 | C51 | M8 | C12 | -45,529307 | -72,029745 | 715,3 m | KF718564 | KF718438 |
| | *N. pumilio* middle-aged forest 2 | C52 | M6 | C1 | -45,529927 | -72,030522 | 686,1 m | KF718565 | KF718439 |
| Karukinka | *N. pumilio* young forest | K1 | M5 | C2 | -54,139677 | -68,710203 | 186,6 m | **KC514744** | **KC514624** |
| | *N. pumilio* young forest | K2 | M5 | C2 | -54,139630 | -68,709712 | 171,6 m | **KC514745** | **KC514625** |
| | *N. pumilio* young forest | K3 | M5 | C2 | -54,139668 | -68,709812 | 168,7 m | **KC514746** | **KC514626** |
| | *N. pumilio* young forest | K4 | M5 | C2 | -54,139758 | -68,709845 | 197,2 m | **KC514747** | **KC514627** |
| | *N. pumilio* young forest | K5 | M4 | C3 | -54,139775 | -68,710757 | 182,4 m | **KC514748** | **KC514628** |
| | *N. pumilio* young forest | K6 | M1 | C2 | -54,139718 | -68,709743 | 185,1 m | **KC514749** | **KC514629** |
| | *N. pumilio* young forest | K7 | M5 | C2 | -54,139782 | -68,709787 | 178,3 m | **KC514750** | **KC514630** |
| | *N. pumilio* young forest | K8 | M4 | C3 | -54,139773 | -68,710823 | 197,5 m | **KC514751** | **KC514631** |
| | *N. pumilio* young forest | K9 | M4 | C3 | -54,139792 | -68,710827 | 196,2 m | **KC514752** | **KC514632** |
| | *N. pumilio* young forest | K10 | M4 | C3 | -54,139717 | -68,710762 | 190,8 m | **KC514753** | **KC514633** |

| Sampling site information | | | | | Samples information (geo-reference data) | | | Sequences information (accession numbers) | |
|---|---|---|---|---|---|---|---|---|---|
| **Site** | **Environmental Context** | **Sample** | ***Ms* OTU** | ***Cs* OTU** | **Latitude (S)** | **Longitude (W)** | **Altitude** | **28S rRNA gene*** | **16S rRNA gene*** |
| | *N. pumilio* young forest | K11 | M2 | C6 | -54,139712 | -68,710878 | 194,9 m | **KC514754** | **KC514634** |
| | *N. pumilio* young forest | K12 | M1 | C2 | -54,139690 | -68,709768 | 187,5 m | **KC514755** | **KC514635** |
| | *N. pumilio* young forest | K13 | M2 | C6 | -54,139718 | -68,709785 | 191,4 m | **KC514756** | **KC514636** |
| | *N. pumilio* young forest | K14 | M2 | C6 | -54,139678 | -68,709775 | 189,2 m | **KC514757** | **KC514637** |
| | *N. pumilio* young forest | K15 | M5 | C2 | -54,139730 | -68,709760 | 187,0 m | **KC514758** | **KC514638** |
| | *N. pumilio* young forest | K16 | M2 | C6 | -54,139638 | -68,709830 | 181,5 m | **KC514759** | **KC514639** |
| | *N. pumilio* young forest | K17 | M2 | C6 | -54,139633 | -68,709872 | 183,0 m | **KC514760** | **KC514640** |
| | *N. pumilio* young forest | K18 | M2 | C6 | -54,139642 | -68,709863 | 183,0 m | **KC514761** | **KC514641** |
| | *N. pumilio* young forest | K19 | M2 | C6 | -54,139657 | -68,709793 | 186,6 m | **KC514762** | **KC514642** |
| | *N. pumilio* young forest | K20 | M2 | C6 | -54,139705 | -68,709818 | 190,4 m | **KC514763** | **KC514643** |
| | *N. pumilio* mature forest | K21 | M1 | C15 | -54,126923 | -68,709487 | 169,3 m | **KC514764** | **KC514644** |
| | *N. pumilio* mature forest | K22 | M5 | C15 | -54,126932 | -68,709418 | 171,3 m | **KC514765** | **KC514645** |
| | *N. pumilio* mature forest | K23 | M5 | C2 | -54,126787 | -68,709200 | 170,8 m | **KC514766** | **KC514646** |
| | *N. pumilio* mature forest | K24 | M6 | C14 | -54,126657 | -68,709040 | 168,8 m | **KC514767** | **KC514647** |
| | *N. pumilio* mature forest | K25 | M8 | C10 | -54,126602 | -68,709053 | 162,9 m | **KC514768** | **KC514648** |
| | *N. pumilio* mature forest | K26 | M5 | C2 | -54,126725 | -68,709285 | 161,9 m | **KC514769** | **KC514649** |
| | *N. pumilio* mature forest | K27 | M1 | C2 | -54,126743 | -68,709635 | 169,2 m | **KC514770** | **KC514650** |
| | *N. pumilio* mature forest | K28 | M6 | C2 | -54,126820 | -68,709563 | 170,1 m | **KC514771** | **KC514651** |
| | *N. pumilio* mature forest | K29 | M5 | C2 | -54,127087 | -68,709597 | 169,0 m | **KC514772** | **KC514652** |
| | *N. pumilio* mature forest | K30 | M1 | C2 | -54,127133 | -68,709558 | 168,8 m | **KC514773** | **KC514653** |
| | *N. pumilio* mature forest | K31 | M1 | C2 | -54,127107 | -68,709558 | 169,2 m | **KC514774** | **KC514654** |
| | *N. pumilio* mature forest | K32 | M1 | C2 | -54,127305 | -68,709583 | 171,3 m | **KC514775** | **KC514655** |
| | *N. pumilio* mature forest | K33 | M5 | C2 | -54,127305 | -68,709583 | 171,3 m | **KC514776** | **KC514656** |
| | *N. pumilio* mature forest | K34 | M5 | C2 | -54,127153 | -68,709772 | 161,4 m | **KC514777** | **KC514657** |
| | *N. pumilio* mature forest | K35 | M5 | C2 | -54,127148 | -68,709327 | 159,6 m | **KC514778** | **KC514658** |
| | *N. pumilio* mature forest | K36 | M5 | C2 | -54,127113 | -68,709312 | 159,6 m | **KC514779** | **KC514659** |
| | *N. pumilio* mature forest | K37 | M6 | C14 | -54,127083 | -68,709270 | 180,6 m | **KC514780** | **KC514660** |
| | *N. pumilio* mature forest | K38 | M3 | C8 | -54,126927 | -68,709337 | 153,0 m | **KC514781** | **KC514661** |
| | *N. pumilio* mature forest | K39 | M4 | C7 | -54,127305 | -68,709613 | 198,8 m | **KC514782** | **KC514662** |
| | *N. pumilio* mature forest | K40 | M1 | C15 | -54,127270 | -68,709775 | 178,1 m | **KC514783** | **KC514663** |
| | Grassland | K41 | M4 | C3 | -54,126535 | -68,709005 | 146,0 m | **KC514784** | **KC514664** |
| | Grassland | K42 | M4 | C3 | -54,126588 | -68,708953 | 147,2 m | **KC514785** | **KC514665** |

| Sampling site information | | | | | Samples information (geo-reference data) | | | Sequences information (accession numbers) | |
|---|---|---|---|---|---|---|---|---|---|
| **Site** | **Environmental Context** | **Sample** | ***Ms* OTU** | ***Cs* OTU** | **Latitude (S)** | **Longitude (W)** | **Altitude** | **28S rRNA gene*** | **16S rRNA gene*** |
| | Grassland | K43 | M4 | C4 | -54,126577 | -68,708882 | 145,0 m | **KC514786** | **KC514666** |
| | Grassland | K44 | M4 | C4 | -54,126430 | -68,708863 | 152,1 m | **KC514787** | **KC514667** |
| | Grassland | K45 | M4 | C7 | -54,126428 | -68,708597 | 154,0 m | **KC514788** | **KC514668** |
| | Grassland | K46 | M4 | C14 | -54,126385 | -68,708490 | 160,0 m | **KC514789** | **KC514669** |
| | Grassland | K47 | M4 | C3 | -54,126358 | -68,708532 | 159,0 m | **KC514790** | **KC514670** |
| | Grassland | K48 | M4 | C7 | -54,126363 | -68,708618 | 158,6 m | **KC514791** | **KC514671** |
| | Grassland | K49 | M4 | C7 | -54,126367 | -68,708592 | 157,8 m | **KC514792** | **KC514672** |
| | Grassland | K50 | M4 | C7 | -54,126322 | -68,708813 | 154,1 m | **KC514793** | **KC514673** |
| | Grassland | K51 | M4 | C7 | -54,126292 | -68,708772 | 155,9 m | **KC514794** | **KC514674** |
| | Grassland | K52 | M4 | C7 | -54,126317 | -68,708877 | 152,4 m | **KC514795** | **KC514675** |
| | Grassland | K53 | M4 | C7 | -54,126352 | -68,708983 | 151,0 m | **KC514796** | **KC514676** |
| | Grassland | K54 | M4 | C7 | -54,126227 | -68,708912 | 152,4 m | **KC514797** | **KC514677** |
| | Grassland | K55 | M4 | C7 | -54,126258 | -68,708945 | 152,3 m | **KC514798** | **KC514678** |
| | Grassland | K56 | M4 | C7 | -54,126237 | -68,708807 | 153,6 m | **KC514799** | **KC514679** |
| | Grassland | K57 | M4 | C7 | -54,126193 | -68,708778 | 156,5 m | **KC514800** | **KC514680** |
| | Grassland | K58 | M4 | C7 | -54,126187 | -68,708762 | 157,3 m | **KC514801** | **KC514681** |
| | Grassland | K59 | M4 | C7 | -54,126265 | -68,708748 | 152,1 m | **KC514802** | **KC514682** |
| | Grassland | K60 | M4 | C7 | -54,126247 | -68,708725 | 149,9 m | **KC514803** | **KC514683** |
| Navarino | *N. pumilio* young forest | N1 | M8 | C10 | -54,939233 | -67,602461 | 39,4 m | KF718581 | KF718455 |
| | *N. pumilio* young forest | N2 | M8 | C10 | -54,939147 | -67,602489 | 34,8 m | KF718582 | KF718456 |
| | *N. pumilio* young forest | N3 | M8 | C11 | -54,939033 | -67,602642 | 33,1 m | KF718583 | KF718457 |
| | *N. pumilio* young forest | N4 | M8 | C11 | -54,938972 | -67,603481 | 26,4 m | KF718584 | KF718458 |
| | *N. pumilio* young forest | N5 | M8 | C10 | -54,939644 | -67,602911 | nd | KF718585 | KF718459 |
| | *N. pumilio* young forest | N6 | M7 | C14 | -54,939061 | -67,603669 | 48,1 m | KF718586 | KF718460 |
| | *N. pumilio* young forest | N7 | M8 | C11 | -54,939069 | -67,603250 | 14,0 m | KF718587 | KF718461 |
| | *N. pumilio* young forest | N8 | M8 | C10 | -54,939278 | -67,604058 | 87,8 m | KF718588 | KF718462 |
| | *N. pumilio* young forest | N9 | M7 | C14 | -54,939394 | -67,603911 | 44,8 m | KF718589 | KF718463 |
| | *N. pumilio* young forest | N10 | M8 | C10 | -54,939194 | -67,604011 | 2,8 m | KF718590 | KF718464 |
| | *N. pumilio* young forest | N11 | M8 | C10 | -54,939122 | -67,602331 | 49,3 m | KF718591 | KF718465 |
| | *N. pumilio* young forest | N12 | M8 | C11 | -54,939181 | -67,601972 | 63,9 m | KF718592 | KF718466 |
| | *N. pumilio* young forest | N13 | M8 | C10 | -54,939033 | -67,602719 | 2,6 m | KF718593 | KF718467 |
| | *N. pumilio* young forest | N14 | M8 | C11 | -54,938894 | -67,602228 | 27,7 m | KF718594 | KF718468 |

| Sampling site information | | | | | Samples information (geo-reference data) | | | Sequences information (accession numbers) | |
|---|---|---|---|---|---|---|---|---|---|
| **Site** | **Environmental Context** | **Sample** | ***Ms* OTU** | ***Cs* OTU** | **Latitude (S)** | **Longitude (W)** | **Altitude** | **28S rRNA gene*** | **16S rRNA gene*** |
| | *N. pumilio* young forest | N15 | M8 | C10 | -54,938847 | -67,603111 | nd | KF718595 | KF718469 |
| | *N. pumilio* young forest | N16 | M8 | C10 | -54,938844 | -67,601986 | 72,8 m | KF718596 | KF718470 |
| | *N. pumilio* young forest | N17 | M8 | C12 | -54,938781 | -67,602211 | 64,3 m | KF718597 | KF718471 |
| | *N. pumilio* young forest | N18 | M7 | C14 | -54,938853 | -67,602525 | 20,2 m | KF718598 | KF718472 |
| | *N. pumilio* young forest | N19 | M8 | C10 | -54,938806 | -67,602172 | 34,5 m | KF718599 | KF718473 |
| | *N. pumilio* young forest | N20 | M8 | C10 | -54,938969 | -67,602319 | 24,6 m | KF718600 | KF718474 |
| | *N. pumilio* mature forest | N21 | M8 | C11 | -54,948239 | -67,656414 | 75,2 m | KF718601 | KF718475 |
| | *N. pumilio* mature forest | N22 | M8 | C10 | -54,948325 | -67,656456 | 103,3 m | KF718602 | KF718476 |
| | *N. pumilio* mature forest | N23 | M8 | C11 | -54,948603 | -67,655883 | 95,7 m | KF718603 | KF718477 |
| | *N. pumilio* mature forest | N24 | M8 | C10 | -54,948211 | -67,654447 | 119,6 m | KF718604 | KF718478 |
| | *N. pumilio* mature forest | N25 | M8 | C11 | -54,948289 | -67,655128 | 121,1 m | KF718605 | KF718479 |
| | *N. pumilio* mature forest | N26 | M8 | C11 | -54,948264 | -67,654931 | 86,5 m | KF718606 | KF718480 |
| | *N. pumilio* mature forest | N27 | M8 | C11 | -54,948322 | -67,654219 | 122,1 m | KF718607 | KF718481 |
| | *N. pumilio* mature forest | N28 | M8 | C11 | -54,948328 | -67,654889 | 116,5 m | KF718608 | KF718482 |
| | *N. pumilio* mature forest | N29 | M8 | C11 | -54,948464 | -67,654628 | 139,0 m | KF718609 | KF718483 |
| | *N. pumilio* mature forest | N30 | M8 | C11 | -54,948081 | -67,653619 | 33,8 m | KF718610 | KF718484 |
| | *N. pumilio* mature forest | N31 | M8 | C11 | -54,948425 | -67,655694 | 9,2 m | KF718611 | KF718485 |
| | *N. pumilio* mature forest | N32 | M8 | C11 | -54,948381 | -67,653761 | 164,8 m | KF718612 | KF718486 |
| | *N. pumilio* mature forest | N33 | M8 | C11 | -54,948414 | -67,654653 | 113,2 m | KF718613 | KF718487 |
| | *N. pumilio* mature forest | N34 | M8 | C12 | -54,948706 | -67,653544 | 74,1 m | KF718614 | KF718488 |
| | *N. pumilio* mature forest | N35 | M8 | C11 | -54,948297 | -67,654128 | 135,2 m | KF718615 | KF718489 |
| | *N. pumilio* mature forest | N36 | M8 | C11 | -54,948611 | -67,652719 | 51,2 m | KF718616 | KF718490 |
| | *N. pumilio* mature forest | N37 | M8 | C12 | -54,948636 | -67,647950 | 31,3 m | KF718617 | KF718491 |
| | *N. pumilio* mature forest | N38 | M8 | C12 | -54,948422 | -67,648056 | 45,8 m | KF718618 | KF718492 |
| | *N. pumilio* mature forest | N39 | M8 | C12 | -54,948369 | -67,647856 | 49,7 m | KF718619 | KF718493 |
| | *N. pumilio* mature forest | N40 | M8 | C12 | -54,948106 | -67,648117 | 79,9 m | KF718620 | KF718494 |
| | Grassland | N41 | M1 | C7 | -54,942072 | -67,625772 | 33,1 m | KF718621 | KF718495 |
| | Grassland | N42 | M1 | C7 | -54,942019 | -67,625511 | 34,0 m | KF718622 | KF718496 |
| | Grassland | N43 | M4 | C3 | -54,941969 | -67,625061 | 29,5 m | KF718623 | KF718497 |
| | Grassland | N44 | M1 | C7 | -54,940117 | -67,621767 | 21,4 m | KF718624 | KF718498 |
| | Grassland | N45 | M1 | C14 | -54,939853 | -67,621586 | 20,5 m | KF718625 | KF718499 |
| | Grassland | N46 | M1 | C14 | -54,939633 | -67,621469 | 65,5 m | KF718626 | KF718500 |

| Sampling site information | | | | | Samples information (geo-reference data) | | | Sequences information (accession numbers) | |
|---|---|---|---|---|---|---|---|---|---|
| **Site** | **Environmental Context** | **Sample** | ***Ms* OTU** | ***Cs* OTU** | **Latitude (S)** | **Longitude (W)** | **Altitude** | **28S rRNA gene*** | **16S rRNA gene*** |
| | Grassland | N47 | M1 | C13 | -54,941869 | -67,660394 | 8,8 m | KF718627 | KF718501 |
| | Grassland | N48 | M4 | C3 | -54,941814 | -67,661047 | 23,6 m | KF718628 | KF718502 |
| | Grassland | N49 | M1 | C7 | -54,941811 | -67,660989 | 24,5 m | KF718629 | KF718503 |
| | Grassland | N50 | M4 | C3 | -54,941894 | -67,661139 | 28,0 m | KF718630 | KF718504 |
| | Grassland | N51 | M4 | C3 | -54,941672 | -67,661214 | 35,7 m | KF718631 | KF718505 |
| | Grassland | N52 | M5 | C1 | -54,941883 | -67,661442 | 17,9 m | KF718632 | KF718506 |
| | Grassland | N53 | M4 | C9 | -54,941839 | -67,660272 | 22,2 m | KF718633 | KF718507 |
| | Grassland | N54 | M1 | C14 | -54,939136 | -67,601675 | 46,1 m | KF718634 | KF718508 |
| | Grassland | N55 | M4 | C14 | -54,939006 | -67,601806 | 47,4 m | KF718635 | KF718509 |
| | Grassland | N56 | M1 | C14 | -54,939175 | -67,601947 | 49,4 m | KF718636 | KF718510 |
| | Grassland | N57 | M4 | C14 | -54,939308 | -67,602158 | 45,6 m | KF718637 | KF718511 |
| | Grassland | N58 | M4 | C14 | -54,939269 | -67,602325 | 39,0 m | KF718638 | KF718512 |
| | Grassland | N59 | M4 | C14 | -54,939289 | -67,602439 | 39,1 m | KF718639 | KF718513 |
| | Grassland | N60 | M4 | C14 | -54,939378 | -67,602478 | 42,0 m | KF718640 | KF718514 |
| Deception | volcanic hillside | D1 | M2 | C14 | -62,972691 | -60,575246 | 29,9 m | KF718566 | KF718440 |
| | volcanic hillside | D2 | M2 | C14 | -62,972658 | -60,57515 | 28,1 m | KF718567 | KF718441 |
| | volcanic hillside | D3 | M2 | C14 | -62,97262 | -60,575316 | 23,3 m | KF718568 | KF718442 |
| | volcanic hillside | D4 | M2 | C14 | -62,97264 | -60,575283 | 22,9 m | KF718569 | KF718443 |
| | volcanic hillside | D5 | M2 | C14 | -62,972666 | -60,57537 | 23,6 m | KF718570 | KF718444 |
| | volcanic hillside | D6 | M2 | C14 | -62,972765 | -60,575593 | 24,2 m | KF718571 | KF718445 |
| | volcanic hillside | D7 | M2 | C14 | -62,972758 | -60,575615 | 23,5 m | KF718572 | KF718446 |
| | volcanic hillside | D8 | M2 | C14 | -62,972756 | -60,575648 | 21,6 m | KF718573 | KF718447 |
| | volcanic hillside | D9 | M2 | C14 | -62,972738 | -60,575691 | 22,7 m | KF718574 | KF718448 |
| | volcanic hillside | D10 | M2 | C14 | -62,973043 | -60,575723 | 16,6 m | KF718575 | KF718449 |
| | volcanic hillside | D11 | M2 | C14 | -62,973046 | -60,576206 | 16,2 m | KF718576 | KF718450 |
| | volcanic hillside | D12 | M2 | C14 | -62,973048 | -60,576203 | 16,2 m | KF718577 | KF718451 |
| | volcanic hillside | D13 | M2 | C14 | -62,973055 | -60,576195 | 15,5 m | KF718578 | KF718452 |
| | volcanic hillside | D14 | M2 | C14 | -62,973055 | -60,576193 | 15,5 m | KF718579 | KF718453 |
| | volcanic hillside | D15 | M2 | C14 | -62,973056 | -60,576193 | 15,4 m | KF718580 | KF718454 |

*Accession numbers in bold correspond to the sequences from Karukinka published in Ramírez-Fernández *et al.* (41).
